# Optical breakdown of solids by few-cycle laser pulses

**DOI:** 10.1038/s41598-017-18624-z

**Published:** 2018-01-29

**Authors:** P. A. Zhokhov, A. M. Zheltikov

**Affiliations:** 1 Department of Physics and Astronomy, Texas A&M University, 77843 College Station, TX USA; 2 Russian Quantum Center, 143025 Skolkovo, Moscow Region Russia; 3 Physics Department, International Laser Center, M.V. Lomonosov Moscow State University, 119992 Moscow, Russia; 4 Kazan Quantum Center, A.N. Tupolev Kazan National Research Technical University, 420126 Kazan, Russia; 5 Kurchatov Institute National Research Center, Moscow, 123182 Russia

## Abstract

We show that a broadly accepted criterion of laser-induced breakdown in solids, defining the laser-breakdown threshold in terms of the laser fluence or laser intensity needed to generate a certain fraction of the critical electron density rc within the laser pulse, fails in the case of high-intensity few-cycle laser pulses. Such laser pulses can give rise to subcycle oscillations of electron density *ρ* with peak *ρ* values well above *ρ*_c_ even when the total energy of the laser pulse is too low to induce a laser damage of material. The central idea of our approach is that, instead of the *ρ* = *ρ*_*c*_ ratio, the laser-breakdown threshold connects to the total laser energy coupled to the electron subsystem and subsequently transferred to the crystal lattice. With this approach, as we show in this work, predictions of the physical model start to converge to the available experimental data.

## Introduction

Laser-induced breakdown of solid materials has been a subject of in-depth research since the invention of lasers^1,2^. In the era of rapidly progressing laser sources of extremely short and broadband optical field waveforms^3,4^, understanding the regimes and scenarios of laser-induced breakdown, as well as the available parameter space for a reversible photoionization-assisted control of optical properties of solids is central for emerging petahertz optoelectronic technologies^5,6^, nonlinear-optical bioimaging^7,8^, short-pulse laser surgery^9,10^, laser micromachining^11^, and compression of high-peak-power ultrashort laser pulses in transparent solids^12–14^.

Systematic experimental studies of optical breakdown and laser-induced damage, performed within more than five decades, have revealed distinctly different physical scenarios of optical breakdown induced by laser pulses of broadly varying intensities, fluences, and pulse widths^15–17^. These studies helped identify a broad range of physical processes contributing to laser-induced breakdown^18^, including field-induced and avalanche ionization, nonlinear dynamics of a laser beam, plasma effects, radiation absorption by impurity and defect states, as well as collisional dynamics, diffusion, and recombination of free carriers^9^.

While the specific regime of laser-induced breakdown can depend on all the above-listed factors, ionization dynamics and the related buildup of free-carrier density always play a central role in laser breakdown, providing a mechanism whereby the laser field is coupled to a material. This fact is recognized by a broadly accepted criterion of laser-induced breakdown^9,18–25^ that defines the laser breakdown threshold in terms of the laser fluence or laser intensity needed to generate a certain fraction of the critical electron density within the laser pulse. This criterion has proven to be useful in a broad range of pulse widths, offering a powerful tool for the analysis of a laser breakdown by pico- and femtosecond light pulses and helping understand a variety of related laser–matter interaction phenomena in a broad class of solid materials and systems, including laser-induced filamentation^23–26^, laser micromachining^11^, laser biomedicine^9,10^, supercontinuum generation^24,25,27^, and compression^12,13,28,29^ of high-power laser pulses in solids.

Here, we show, however, that this broadly accepted approach to assessing the role of laser-induced breakdown in solids fails in the case of high-intensity few-cycle laser pulses. Such laser pulses can give rise to subcycle oscillations of electron density *ρ* with peak *ρ* values well above the critical electron density *ρ*_*c*_ without inducing a laser damage of material. Analysis of rapidly varying ionization-induced refraction and loss in solids along with an hierarchy of energy transfer processes, occurring on drastically different time scales suggests that the laser-breakdown threshold of solids rather connects to the laser energy absorbed within a unit volume of a solid and subsequently transferred to the crystal lattice.

## Subcycle Ionization Dynamics

We first demonstrate the difficulties that the standard criterion of laser-induced breakdown encounters when applied to a laser damage of a solid by a very short high-intensity laser pulse. To this end, we consider a transparent dielectric with a dispersion relation

1 $$ {\mathcal E} (\overrightarrow{p})={ {\mathcal E} }_{g}+{\rm{\Delta }}-\frac{{\rm{\Delta }}}{D}\sum _{i=1}^{D}\,\cos (\frac{{p}_{i}d}{\hslash })$$

where ${ {\mathcal E} }_{g}$ is the band gap, Δ is the band width, *D* is the number of spatial dimensions, *p*_*i*_ is the *i*th Cartesian component of the momentum $\overrightarrow{P}$, and *d* is the lattice constant.

The density of conduction-band (CB) electrons induced in such a material by an ultrashort laser pulse is given by^30,31^

2 $$\rho (t)=|{\mathscr{N}}{|}^{2}{\int }_{-\infty }^{t}d{t}_{1}{\int }_{-\infty }^{t}d{t}_{2}\overrightarrow{E}({t}_{1})\cdot \overrightarrow{E}({t}_{2})G({t}_{1},{t}_{2})$$

where $\overrightarrow{E}$(*t*) is the electric field, $|{\mathscr{N}}{|}^{2}$ is a normalization constant,

3 $$G({t}_{1},{t}_{2})={e}^{i\frac{{ {\mathcal E} }_{g}+{\rm{\Delta }}}{\hslash }({t}_{2}-{t}_{1})}\prod _{j=1}^{D}{J}_{0}({{\rm{\Phi }}}_{j}({t}_{2},{t}_{1})),$$

4 $${{\rm{\Phi }}}_{j}({t}_{2},{t}_{1})={\int }_{{t}_{1}}^{{t}_{2}}dt^{\prime} {e}^{i\frac{e{A}_{j}(t^{\prime} )d}{\hslash }},$$

and *A*_*j*_(*t*) are the components of the vector potential

5 $$\overrightarrow{A}(t)={\int }_{-\infty }^{t}dt^{\prime} E(t^{\prime} )$$

along the crystal axes.

**Figure 1:**
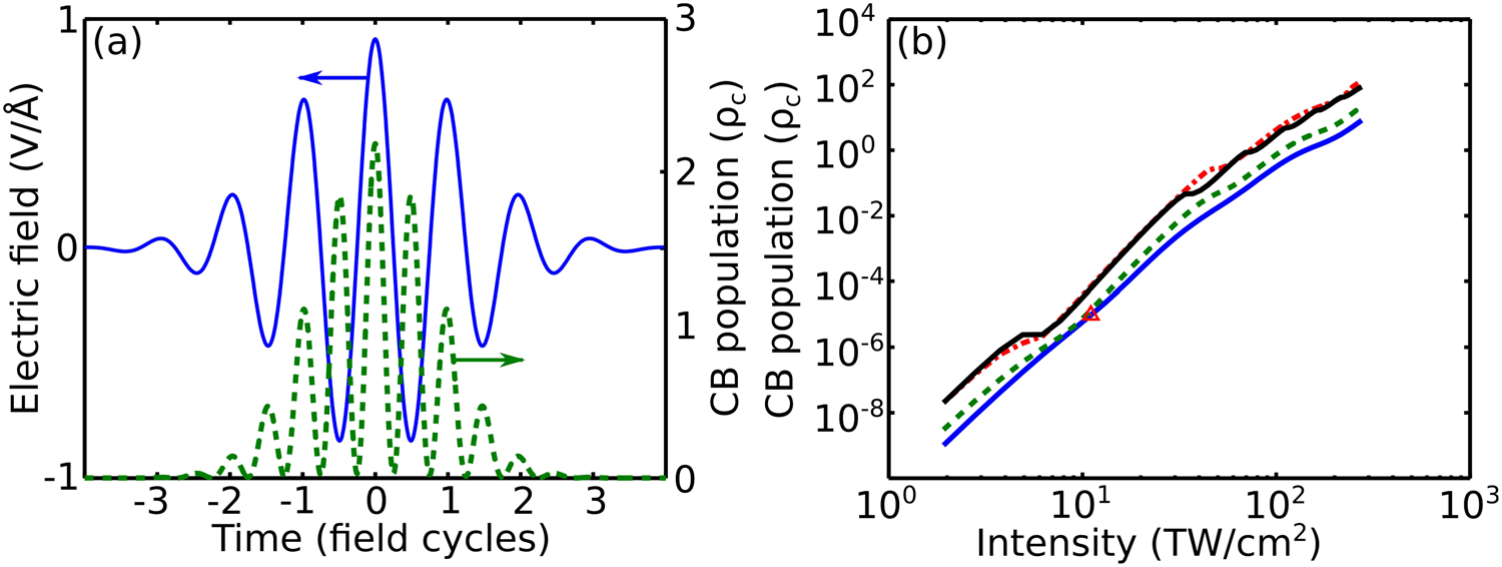
(**a**) The dynamics of conduction-band population (green dashed line) induced by a Gaussian laser pulse with a central wavelength *λ*_0_ = 800 nm, a peak intensity *I*_0_ = 10 TW/cm^2^, and a pulse width *τ* = 5.3 fs (shown by the blue solid line) in fused silica (${ {\mathcal E} }_{g}=9$ eV, ${\rm{\Delta }}=0.6{ {\mathcal E} }_{g}$, and *d* = 0.2 nm). (**b**) The conduction-band population in the wake of the laser pulse with *λ*_0_ = 800 nm as a function of the peak intensity *I*_0_ for *τ* = 5.3 fs (blue solid line), 13 fs (green dashed line), and 80 fs (red dash–dotted line). The conduction-band population calculated using the Keldysh formalism of Eq. (7) for laser pulses with *τ* = 80 fs is shown by the black line.

In Fig. 1(a), we show the electron density *ρ*(*t*) induced by a Gaussian laser pulse with a central wavelength *λ*_0_ = 800 nm, a pulse width *τ* = 5.3 fs (corresponding to two field cycles), and a peak intensity *I* = 10 TW/cm^2^ in fused silica (${ {\mathcal E} }_{g}=9$ eV, ${\rm{\Delta }}=0.6{ {\mathcal E} }_{g}$, and *d* = 0.2 nm). The electron density *ρ*(*t*) induced by such a field waveform is seen to display a characteristic oscillatory behavior, following oscillations of the field intensity in the driver pulse^30^. As one of the most striking results, we find that the peak electron density achieved as a part of this subcycle ionization dynamics [Fig. 1(a)] is noticeably higher than the critical electron density

6 $${\rho }_{c}=\frac{{m}^{\ast }{\omega }^{2}}{4\pi {e}^{2}},$$

where *e* is the electron charge, m* is the effective electron mass, and *ω* is the frequency.

We are clearly running into a problem here, trying to apply the standard criterion of optical breakdown to this case. On the one hand, the instantaneous electron density reaches levels way above *ρ*_*c*_ as a part of its oscillatory dynamics on the subcycle time scale. On the other hand, the electron density in the wake of the laser pulse is up to six orders of magnitude lower than *ρ*_*c*_ [the red triangle in Fig. 1(b)].

**Figure 2:**
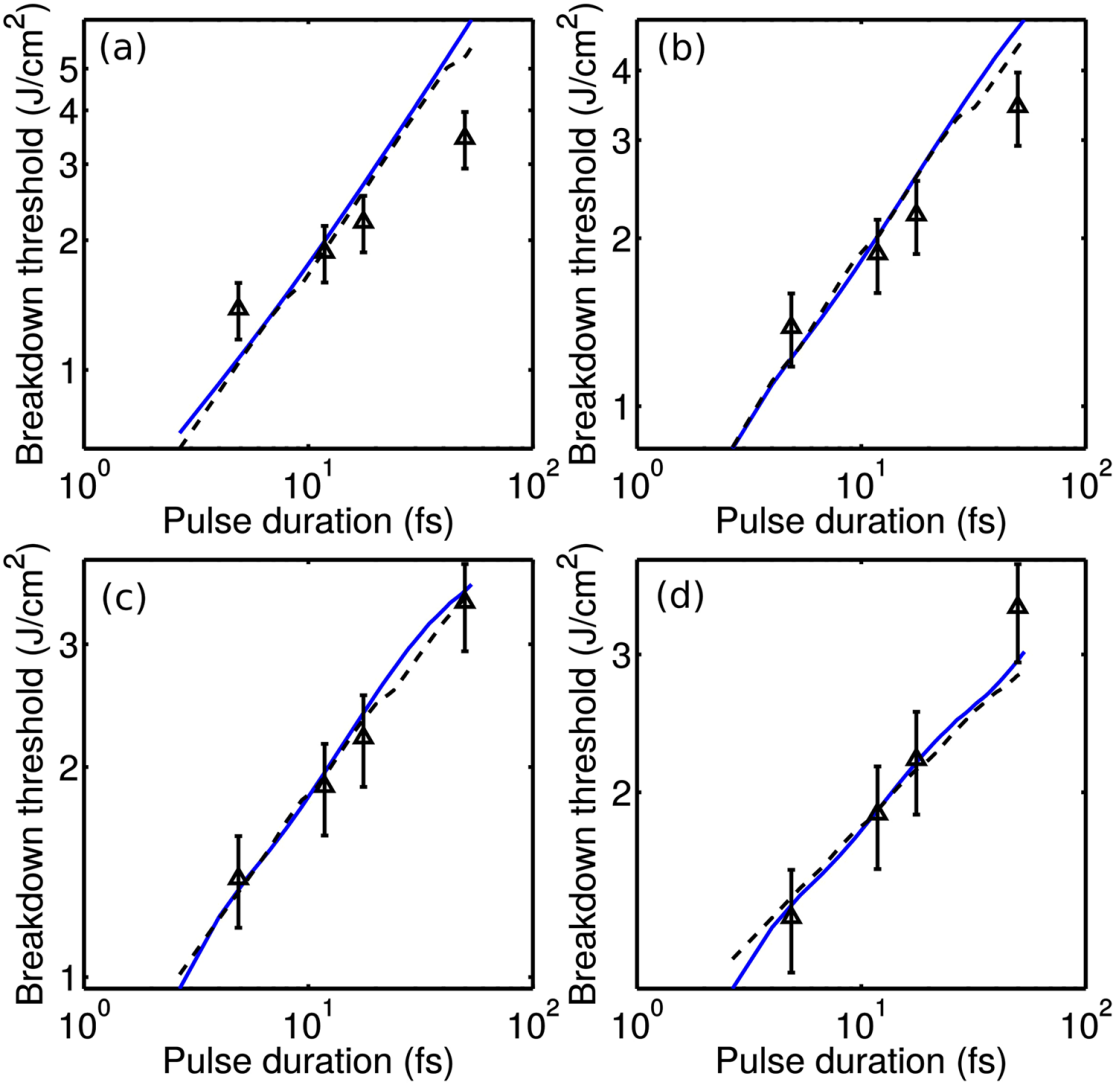
The threshold fluence *F*_*th*_ corresponding to an optical breakdown of fused silica by a laser pulse with *λ*_0_ = 800 nm as a function of the laser pulse width: (triangles with error bars) measurements by Lenzner *et al*.^17^, (green dashed line) calculations using the rate equation (7) with the Keldysh formula for the photoionization rate, and (solid blue line) calculations using the model of Eqs (8, 20, 31) with *σ* = 0 (**a**), 2 × 10^−18^ cm^−2^ (**b**), 5 × 10^−18^ cm^−2^ (**c**), and 10^−17^ cm^−2^ (**d**); *ρ*_*th*_ = 10^21^ cm^−3^ (a), 3.4 × 10^21^ cm^−3^ (**b**), 1.2 × 10^22^ cm^−3^ (**c**), and 1.5 × 10^23^ cm^−3^ (**d**); *w*_*th*_ = 3 kJ/cm^3^ (**a**), 6.8 kJ/cm^3^ (**b**), 21 kJ/cm^3^ (**c**), and 160 kJ/cm^3^ (**d**).

In Fig. 2, we plot the threshold fluence *F*_*th*_ corresponding to an optical breakdown of fused silica by a laser pulse with *λ*_0_ = 800 nm measured by Lenzner *et al*.^17^ as a function of the laser pulse width. Experiments clearly show that no laser breakdown occurs when fused silica is irradiated by laser pulses with the above-specified parameters. Indeed, for laser pulses with *I* ≈ 10 TW/cm^2^ and *τ* ≈ 5 fs, the energy fluence is *F* ≈ 0.05 J/cm^2^, which is about 30 times lower than the typical fluence *F*_*th*_ needed^17^ for ≈5-fs laser pulses to induce optical breakdown in fused silica (*F*_*th*_ ≈ 1.3 J/cm^2^ for *τ* ≈ 5 fs in Fig. 2).

The laser-breakdown criterion formulated in terms of *ρ*(*t*) and *ρ*_*c*_ thus clearly fails to explain the available experimental data for laser breakdown induced by ultrasort laser pulses. Still, to fully explore the potential of the criteria connecting the laser-breakdown threshold to the electron density, we modify the procedure that we use to find the threshold *ρ* values, *ρ*_*th*_. This modified procedure is based on the standard rate equation for the electron density *ρ*(*t*)^17,32,33^,

7 $$\frac{\partial \rho }{\partial t}=W({I}_{p}(t))+\frac{\sigma }{{ {\mathcal E} }_{g}}\rho {I}_{p}(t),$$

where *W*(*I*) is the photoionization rate, *I*_*p*_(*t*) is the temporal envelope of the field intensity with no carrier wave, and *σ* is the avalanche ionization cross section. In writing Eq. (7), we take into consideration that, according to the predictions of quantum kinetic models^34^, confirmed by the available experimental data^35^, the electron–hole plasma produced by photionization of a solid dielectric develops a collective plasma behavior with characteristic dynamic screening on a typical time scale of *τ*_*pl*_ ~ 1/*ω*_*pl*_, where *ω*_*pl*_ is the plasma frequency. Near the threshold of optical breakdown in our system, the time *τ*_*pl*_ is less than 1 fs.

With the photoionization rate *W*(*I*) calculated with the use of the Keldysh theory of photoionization^36^, which involves averaging over the field cycle, Eq. (7) dictates *ρ*(*t*) profiles that monotonically grow within the laser pulse. We can now solve Eq. (7) for a given pulse width *τ* (the abscissa axis in Fig. 2), a Gaussian pulse shape, and a peak intensity corresponding to a given threshold fluence *F*_*th*_ (the ordinate axis in Fig. 2) to find the *ρ* value in the wake of the laser pulse, which we define as *ρ*_*th*_. With the cross section *σ* treated as a fitting parameter, the entire set of experimental results in Fig. 2 can be fitted with a single *ρ*_*th*_ value.

The result of such a fitting-based procedure is quite reasonable. With the momentum transfer collision time *τ*_*c*_ ≈  1.7 fs^37^, the Drude formula yields an estimate *σ* ≈ 7 × 10^−18^ cm^−2^ for *λ*_0_ = 800 nm. Varying *σ* from 2 × 10^−18^ cm^−2^ to 10^−17^ cm^−2^, we achieve the best fit of experimental results in Fig. 2 with *ρ*_*th*_ ranging from 3.4 × 10^21^ cm^−3^ to 1.5 × 10^23^ cm^−3^. While the maximum values of *ρ*_*th*_ in this range [on the order of 10^23^ cm^−3^ in Fig. 2(d)] are, perhaps, unrealistically high, *ρ*_*th*_ values around 2−3 × 10^21^ [Fig. 2(b)] are consistent with a standard estimate on the critical electron density for *λ*_0_ = 800 nm, *ρ*_*c*_ ≈ 2 × 10^21^. However, we arrive at this satisfactory result at a cost of completely ignoring the essential physical features of subcycle ionization dynamics, which dictates electron densities with peak values well above *ρ*_*c*_ (Fig. 1).

**Figure 3:**
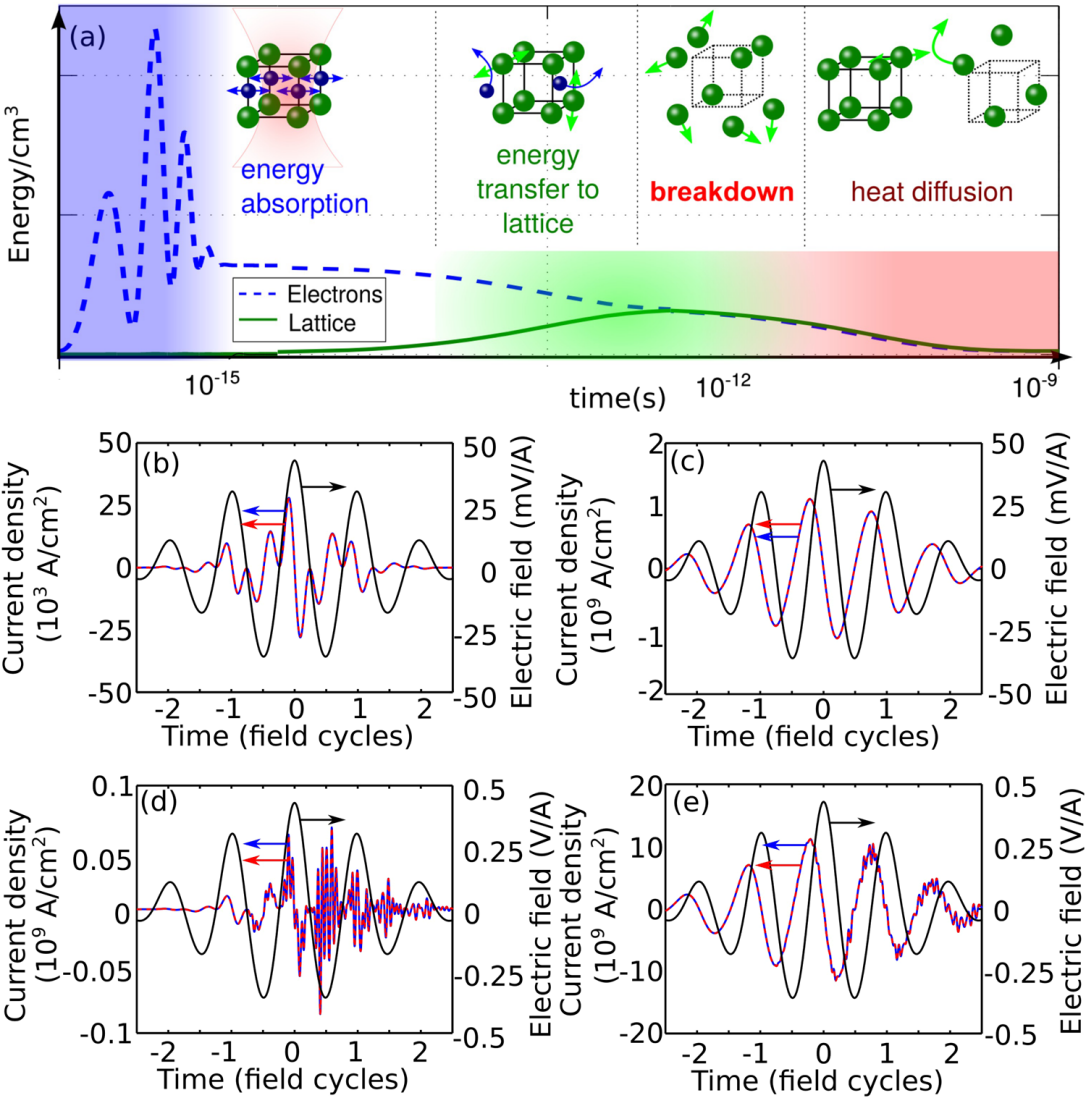
(**a**) Diagram of energy-transfer processes in a solid: (blue dashed line) the energy per unit volume stored in the electronic subsystem, and (green solid line) the energy per unit volume transferred to the lattice. (**b**–**e**) Dynamics of the density of intraband (**b**–**d**) and interband (**c**–**e**) currents induced in diamond by a two-cycle laser pulse with *λ*_0_ = 1.6 *μ*m and the field intensity of (**b**–**c**) 0.02 TW/cm^2^ and (**d**,**e**) 2.2 TW/cm^2^. Calculations were performed using the Schrödinger equation (blue solid line) and the model of Eqs (15, 19) (red dashed line). The electric field of the pulse is shown by the black solid line.

In search for the way out of this difficulty, we resort, in the next section, to the analysis of rapidly varying ionization-induced refraction and loss, governed by the pertinent photocurrents, as well as an hierarchy of energy transfer processes, occurring on drastically different time scales [Fig. 3(a)]. In the case of a few-cycle laser pulse, this sequence of processes starts with ultrafast photoionization and generation of rapidly oscillating photocurrents – the mechanism whereby laser energy is coupled into the electron subsystem of a solid as a part of subcycle photoionization dynamics [shown by blue shading on the subfemtosecond to femtosecond time scale in Fig. 3(a)]. The energy of electron excitation is then transferred, on a much slower time scale, to the crystal lattice [green shading in Fig. 3(a)]. The central idea behind our definition of the optical breakdown threshold is to compare the energy stored in such electron excitations to the energy required to cause melting of the material. With this approach, as we will show below in this paper, the physical models of laser–solid interactions start to converge to the available experimental data on laser-induced breakdown.

## Electrodynamic framework

We start with the analysis of the material relation between the electric displacement $\overrightarrow{D}$(*t*) and the external electric field *E*(*t*) inducing photoionization in a semiconductor:

8 $$\overrightarrow{D}(t)=\varepsilon ({\omega }_{0})\overrightarrow{E}(t)+4\pi \overrightarrow{P}(t\mathrm{)}.$$

Here, *ε*(*ω*_0_) is the dielectric constant of a solid in the absence of the external electric field and $\overrightarrow{P}$(*t*) is the polarization related to the field-induced photocurrent $\overrightarrow{J}$(*t*),

9 $$\overrightarrow{P}(t)={\int }_{-\infty }^{t}\overrightarrow{J}(t^{\prime} )dt^{\prime} .$$

It is physically meaningful^38^ to represent the photocurrent $\overrightarrow{J}$(*t*) as a sum of the intraband (conductivity) and interband (or photoabsorption) terms, $\overrightarrow{J}$^*c*^(*t*) and $\overrightarrow{J}$^*PA*^(*t*):

10 $$\overrightarrow{J}(t)={\overrightarrow{J}}^{c}(t)+{\overrightarrow{J}}^{PA}(t),$$

with

11 $${\overrightarrow{J}}^{c}(t)=e{\int }_{BZ}|\overrightarrow{L}(\overrightarrow{p},t{)|}^{2}\overrightarrow{v}(\overrightarrow{p}+e\overrightarrow{A}(t)){d}^{D}\vec{p},$$

12 $${\overrightarrow{J}}^{PA}(t)\cdot \overrightarrow{E}(t)={\int }_{BZ} {\mathcal E} (\overrightarrow{p}+e\overrightarrow{A}(t))\frac{\partial }{\partial t}|\overrightarrow{L}(\overrightarrow{p},t{)|}^{2}{d}^{D}\vec{p}.$$

Here, ∫_*BZ*_ *d*^*D*^ $\overrightarrow{P}$*;* is the integral over the first Brillouin zone, *D* is the number of spatial dimensions, ${\mathcal E} (\overrightarrow{p})$ is the electron–hole dispersion relation, $\overrightarrow{v}(\overrightarrow{p})=\frac{\partial  {\mathcal E} (\overrightarrow{p})}{\partial \overrightarrow{p}}$ is the velocity corresponding to the momentum $\overrightarrow{P}$, $\overrightarrow{A}={\int }_{-\infty }^{t}\overrightarrow{E}(t^{\prime} )dt^{\prime}$ is the vector potential, and $\overrightarrow{L}$($\overrightarrow{P}$,*t*) is the transition amplitude,

13 $$\overrightarrow{L}(\overrightarrow{p},t)=\frac{{\mathscr{N}}}{{\mathrm{(2}\pi )}^{D}}{\int }_{-\infty }^{t}\overrightarrow{E}(t){e}^{i{\int }_{t^{\prime} }^{t} {\mathcal E} (p+eA(\tau ))d\tau }.$$

The plasma electron density *ρ*(*t*) can now be calculated by integrating the density of conduction-band electrons in $\overrightarrow{P}$:

14 $$\rho (t)={\int }_{BZ}|\overrightarrow{L}(\overrightarrow{p},t{)|}^{2}{d}^{D}\overrightarrow{p}$$

Following ref.^30^, we find

15 $${\overrightarrow{J}}^{c}(t)={\int }_{-\infty }^{t}{\int }_{-\infty }^{t}(\overrightarrow{E}({t}_{1})\cdot \overrightarrow{E}({t}_{2})){\overrightarrow{G}}^{c}(t,{t}_{1},{t}_{2})d{t}_{1}d{t}_{2},$$

and

16 $${\overrightarrow{J}}^{PA}(t)={\int }_{-\infty }^{t}\overrightarrow{E}({t}_{1}){\overrightarrow{G}}^{PA}(t,{t}_{1})d{t}_{1},$$

where ${\overrightarrow{G}}_{c}$ (*t*, *t*_1_, *t*_2_) and *G*^*PA*^(*t*, *t*_1_) are the vectors whose *k*th components are given by

17 $$\begin{array}{rcl}{G}_{k}^{c}(t,{t}_{1},{t}_{2}) & = & |{\mathscr{N}}{|}^{2}\frac{{\rm{\Delta }}}{2iD{ {\mathcal E} }_{g}}{e}^{\frac{i}{\hslash }({ {\mathcal E} }_{g}+\Delta )({t}_{2}-{t}_{1})}{J}_{1}(|{{\rm{\Phi }}}_{k}({t}_{1},{t}_{2})|)\\  &  & \times \,\sin ({\rm{\arg }}({{\rm{\Phi }}}_{k}({t}_{1},{t}_{2}))-{\mu }_{k}(t))\prod _{j\ne k}{J}_{0}(|{{\rm{\Phi }}}_{j}({t}_{1},{t}_{2})|)\end{array}$$

and

18 $$\begin{array}{rcl}{G}^{PA}(t,{t}_{1}) & = & \mathrm{2|}{\mathscr{N}}{|}^{2}\,\cos (\frac{({ {\mathcal E} }_{g}+{\rm{\Delta }})}{\hslash }(t-{t}_{1}))\prod _{j\mathrm{=1}}^{D}{J}_{0}(|{{\rm{\Phi }}}_{j}(t,{t}_{1})|)\\  &  & +\frac{2{\rm{\Delta }}|{\mathscr{N}}{|}^{2}}{D{ {\mathcal E} }_{g}}\,\sin (\frac{({ {\mathcal E} }_{g}+{\rm{\Delta }})}{\hslash }(t-{t}_{1})){J}_{1}(|{{\rm{\Phi }}}_{k}({t}_{1},{t}_{2})|)\\  &  & \times \sum _{k=1}^{D}\,\cos ({\rm{\arg }}({{\rm{\Phi }}}_{k}(t,{t}_{1}))-{\mu }_{k}(t))\prod _{j\ne k}{J}_{0}(|{{\rm{\Phi }}}_{j}({t}_{1},{t}_{2})|)\end{array}$$

Here, *μ*_*k*_(*t*) = $\frac{ed}{\pi \hslash }$ and *A*_*k*_(*t*) is the *k* th component of the vector potential.

The avalanche ionization is included in our model through a modified transition amplitude $\overrightarrow{L}$($\overrightarrow{P}$, *t*):

19 $$\overrightarrow{L}(\overrightarrow{p},t)=\frac{{\mathscr{N}}}{{\mathrm{(2}\pi )}^{D}}{\int }_{-\infty }^{t}\overrightarrow{E}(t){e}^{{\int }_{t^{\prime} }^{t}(i {\mathcal E} (p+eA(\tau ))+\alpha (\tau ))d\tau }$$

where

20 $$\alpha (t)=\frac{\sigma }{{ {\mathcal E} }_{g}}I(t)$$

*I*(*t*) = $\frac{c}{4\pi }|\overrightarrow{{\rm{E}}}(t){|}^{2}$ is the field intensity, and *c* is the speed of light in vacuum.

In Fig. 3(b–e), we compare photocurrent calculations using Eqs (15, 19) with the numerical solution of the Schrödinger equation for a dielectric with the dispersion relation as defined by Eq. (1) with parameters as those of diamond. The inter- and intraband photocurrents calculated with the use of our model are seen to be in a close agreement with the solution of the Schrödinger equation for both low- and high-intensity regimes.

Let us verify now that, in the case of long low-intensity laser pulses, Eqs (8, 19) recover the well-known result for the dielectric function of a weakly ionized medium:

21 $${\varepsilon }_{p}(\omega )=\varepsilon (\omega )-\frac{\rho }{{\rho }_{c}(\omega )}$$

To this end, we assume that the temporal envelope $\overrightarrow{E}$_0_(*t*) of the laser pulse $\overrightarrow{E}$(*t*) = $\overrightarrow{E}$_0_(*t*)cos*ω*_0_*t*, then is slowly varying, so that the central frequency of the laser field is well-defined,

22 $$\frac{d}{dt}\,\mathrm{log}\,{E}_{0}(t)\ll {\omega }_{0}.$$

We further assume that, on the time scale of the field cycle, the electron density can be considered a slowly varying function of time,

23 $$\frac{d}{dt}\,\mathrm{log}\,\rho (t)\ll {\omega }_{0}.$$

The laser field is assumed to be weak, so that

24 $$e|\overrightarrow{A}(t)|\ll \hslash /d.$$

Finally, we assume that all the electron–hole pairs are located near the band center in the momentum space, making sure that the effective-mass approximation holds true,

25 $$|\overrightarrow{L}(\overrightarrow{p},t{)|}^{2}\approx \rho (t)\delta (\overrightarrow{p}\mathrm{)}.$$

Eq. (23) implies that interband transitions are negligible. Indeed, with this condition fulfilled, Eq. (12) yields

26 $${\overrightarrow{J}}^{PA}(t)\approx 0.$$

The total photocurrent is thus given by

27 $$\overrightarrow{J}(t)\approx {\overrightarrow{J}}^{c}(t)=e\overrightarrow{v}(e\overrightarrow{A}(t))\rho (t)\approx \frac{{e}^{2}\overrightarrow{A}(t)}{{m}^{\ast }}\rho (t\mathrm{)}.$$

Polarization $\overrightarrow{P}$(*t*) is then written as

28 $$\overrightarrow{P}(t)=\frac{{e}^{2}}{{m}^{\ast }}{\int }_{-\infty }^{t}\rho (t^{\prime} )dt^{\prime} {\int }_{-\infty }^{t^{\prime} }dt^{\prime\prime} \overrightarrow{E}(t^{\prime\prime} )dt^{\prime\prime} .$$

In the approximation of a slowly varying envelope [Eq. (22)], this expression reduces to

29 $$\overrightarrow{P}(t)\approx -\frac{{e}^{2}}{{m}^{\ast }{\omega }^{2}}\rho (t)\overrightarrow{E}(t)=-4\pi \frac{\rho (t)}{{\rho }_{c}({\omega }_{0})}\overrightarrow{E}(t\mathrm{)}.$$

The displacement $\overrightarrow{D}$(*t*) can now be written as

30 $$\overrightarrow{D}(t)=(\varepsilon ({\omega }_{0})-\frac{\rho (t)}{{\rho }_{c}({\omega }_{0})})\overrightarrow{E}(t)={\varepsilon }_{p}({\omega }_{0})\overrightarrow{E}(t),$$

with *ε*_p_ given by Eq. (21), thus recovering the expression for the dielectric function of a weakly ionized medium.

**Figure 4:**
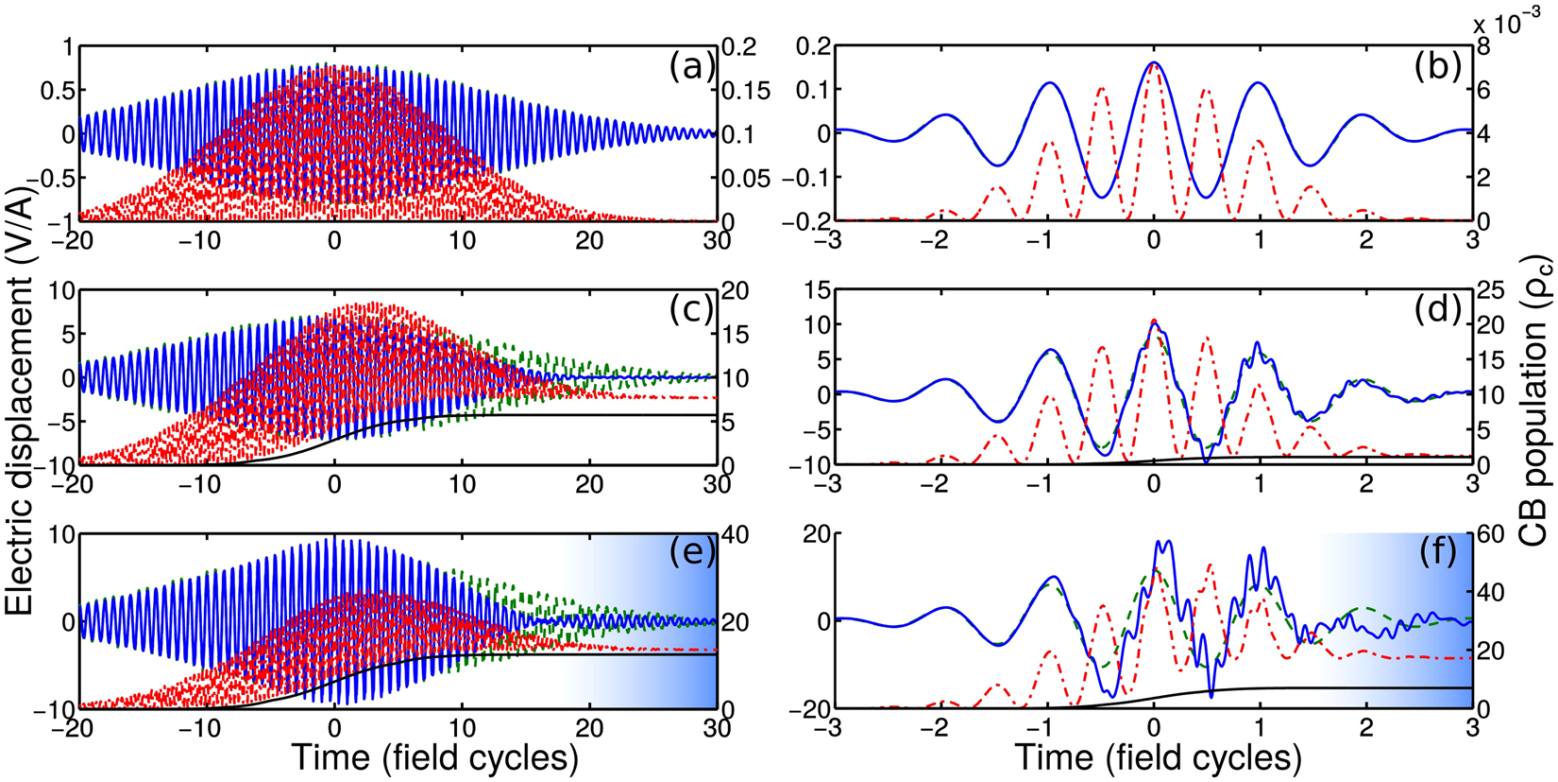
Displacement *D*(*t*) calculated using the full model of Eqs (8, 19) (blue solid curves) and in the approximation of $\overrightarrow{D}$(*t*) = *ε*(*ω*_0_)$\overrightarrow{E}$(*t*) (green dashed line). The red dash–dotted line shows the conduction-band population normalized to the critical electron density *ρ*_*c*_. The black solid line is the electron density calculated using the Keldysh model of photoionization. The pulse width is 30 *T*_0_ (**a**),(**c**),(**e**) and 2 *T*_0_ (**b**),(**d**),(**f**). The peak electric field amplitude is 0.4 V/A (**a**), 0.1 V/A (**b**), 3.3 V/A (**c**), 4 V/A (**d**), 3.8 V/A (**e**), and 5.4 V/A (**f**). The central wavelength of the laser pulse is 800 nm. Parameters of the solid are as those of fused silica.

In Fig. 4, we compare the behavior of $\overrightarrow{D}$(*t*) calculated with the use of the full model of Eqs (8, 19) (blue solid line in Fig. 4) and an approximate formula $\overrightarrow{D}$(*t*) = *ε*(*ω*_0_)$\overrightarrow{E}$(*t*) (green dashed line). In the case of low-intensity laser pulses [Fig. 4(a,b)], when conditions (22–25) are satisfied, the green dashed line, showing $\overrightarrow{D}$(*t*) = *ε*(*ω*_0_), is indistinguishable from the blue line, presenting full-model calculations. The CB electron density in this regime (red dash–dotted line) follows oscillations of the laser field, with the change in the CB population induced by every field half-cycle being vanishingly small, as required by Eq. (23).

At higher laser intensities, when CB population starts to play a significant role [Fig. 4(c–f)], indicating, in violation of Eq. (23), the importance of interband photocurrents, full-model calculations noticeably deviate from the approximation $\overrightarrow{D}$(*t*) = *ε*(*ω*_0_)$\overrightarrow{E}$(*t*). The small amplitude of $\overrightarrow{D}$(*t*) near *t* ≈ 15*T*_0_ (*T*_0_ is the field cycle) in Fig. 4(e) and near *t* ≈ 1.5*T*_0_ in Fig. 4(f) indicates that the electron density induced by the laser pulse is high enough to induce a strong plasma screening of the laser field. As the CB population tends to build up in this regime from the leading edge of the pulse to its trailing edge [red dash–dotted linein Fig. 4(c–f)], plasma screening is dramatically enhanced toward the back of the pulse, in the area shown by blue shading in Fig. 4(e,f). A simple approximate description based on Eqs (21) and (30) clearly fails in this regime.

## Laser-breakdown criterion revisited

We are now in a position to revisit the problem of laser-induced breakdown in a solid. The central idea of our approach is that, instead of the *ρ*/*ρ*_*c*_ ratio, the laser-breakdown threshold should be related to the total laser energy coupled to ultrafast electron excitations in a solid. Using the physical model developed in the previous sections, we can express this energy as

31 $$w={\int }_{-\infty }^{\infty }\overrightarrow{E}(t)\cdot \overrightarrow{J}(t)dt$$

where $\overrightarrow{E}$(*t*) is electric field of the ultrashort pulse.

The product $\overrightarrow{E}$(*t*) ⋅ $\overrightarrow{J}$(*t*) under the integral in Eq. (31) may alter its sign, depending on the phase relation between the driver field $\overrightarrow{E}$(*t*) and the photocurrent $\overrightarrow{J}$(*t*) [see Fig. 3(b–e)]. Unlike *ρ*(*t*), which is rapidly oscillating function of time, whose peak values may substantially exceed *ρ*_*c*_, *w* is an integral parameter, which includes not only the energy transfer from the field to the solid, but also the energy that flows in the opposite direction – from the photocurrent $\overrightarrow{J}$(*t*) back to the field $\overrightarrow{E}$(*t*).

While the laser energy is coupled into the electron subsystem on a very short time scale, through a subcycle photoionization dynamics [blue dashed line and blue shading in Fig. 3(a)], transfer of the energy stored in the electron subsystem to the crystal lattice is a much slower process [green solid line and green shading in Fig. 3(a)]. Indeed, typical times of electron–lattice collisions fall, roughly, within an interval from 1 fs to 1 ps. On this time scale, electrons tend to lose memory of interaction with the laser field, but retain the energy they acquired through such interaction. The energy transfer times are *m*_*e*_/*M* times longer, *m*_*e*_ being the electron mass and *M* being the mass of an ion or an atom in the lattice site. With heat diffusion taking place on pico-to-nanosecond time scale, optical breakdown occurs as the energy transferred to the lattice before this energy dissipates through heat conduction [Fig. 3(a)].

With this physical picture in mind, we attempt to fit the entire set of experimental data for optical breakdown in Fig. 2 with a single *w* value calculated with Eq. (31) and with the avalance ionization cross section σ used as a free parameter. Such fitting curves are shown by solid blue lines in Fig. 2. Remarkably, when no avalanche ionization is included in the model (σ = 0), the theoretical *F*_*th*_(*τ*) dependence calculated with a fixed *w* parameter grows noticeably faster than the *F*_*th*_(*τ*) curve composed of experimental results [Fig. 2(a)]. This finding indicates, in agreement with the experiments of Lenzner *et al*.^17^, the significance of avalance ionization. On the other hand, with the avalance ionization cross section set equal to *σ* = 10^−17^ cm^−2^, i.e., larger than the standard Drude-model estimate for *σ* (*σ* ≈  7 × 10^−18^ cm^−2^ for *λ*_0_ = 800 nm and *τ*_*c*_ ≈  1.7 fs), the *F*_*th*_(*τ*) dependence predicted by calculations with fixed *w* grows noticeably slower than the experimental *F*_*th*_(*τ*) dependence does [Fig. 2(d)].

With *σ* taken equal to 2 × 10^−18^ cm^−2^ and 5 × 10^−18^ cm^−2^ 10^−17^ cm^−2^, a reasonable fit of the experimental *F*_*th*_(*τ*) curve can be achieved [Fig. 2(b,c)] with *w* values of about 7 and 21 kJ/cm ^3^, respectively. This result is consistent with estimates on the energy density required to reach the melting point, which in the case of fused silica typically fall in the range from a few up to tens of kJ/cm^3^ (an illuminating discussion of bond breaking and its role in matter response can be found in refs^39,40^). The results of this analysis are inconclusive as to whether the photon-drag effect^41,42^ and related field-dependent avalanche and cold avalanche processes^43^ can play any measurable role in avalanche ionization in the considered parameter space.

We emphasize once again here that the laser-breakdown problem is considered here in the context of rapidly growing applications of high-peak-power few- and single-cycle laser pulses and their interaction with solids. Those include, but are not limited to petahertz optoelectronic technologies^5,6^, nonlinear-optical bioimaging^7,8^, short-pulse laser surgery^9,10^, laser micromachining^11^, and compression of high-peak-power ultrashort laser pulses in transparent solids^12–14^. Thus, the laser-breakdown problem that we are dealing with in this paper is very different in the underlying physics from many of the short-pulse laser-breakdown- and laser-processing-related problems considered in the extensive earlier literature (see, e.g., refs^24,25,44^ for a review), where the electron density and the related complex refractive index are treated as a slowly varying functions of time and calculated without the inclusion of subcycle ionization dynamics. As a prominent example, a physically insightful model of femtosecond laser ablation proposed by Gamaly *et al*.^45^ provides compact expressions for the threshold of laser ablation of metals and dielectrics applicable for 100-fs pulse widths. In this class of problems, the threshold of laser ablation connects to the Fermi energy in metals or the respective surface binding energy in semiconductors.

The process that we examine in this work is physically different. Here, we deal with a few-cycle laser pulse that penetrates inside a transparent dielectric solid and that can, induce, as the latest experiments show^5,6,46–49^, reversible and irreversible changes in absorption and refractivity of the solid. Laser ablation is out of picture here because the laser beam is focused in such a way that its intensity on the surface of a solid is too low to induce significant photoionization. It is, therefore, photoionization-induced buildup of electron density in the bulk of a transparent solid, rather than the ablation of material from the surface, that eventually leads to optical breakdown in laser–matter interaction geometry considered in this work.

Even more important is that any model that describes laser-induced breakdown, or, for that matter, laser ablation, in terms of plasma refraction and absorption (i.e., the plasma complex refractive index) expressed through the slowly varying electron density calculated using the standard expressions for the photoionization rates averaged over the field cycle, becomes inapplicable in the regime of high-intensity few-cycle pulses, where the subcycle dynamics of photoionization starts to play a prominent role. In this regime, the peak *ρ* values, achieved within a few central half-cycles of the laser field, can significantly exceed, as our analysis shows (Figs 1a, 4b,d,f), the critical electron density *ρ*_*c*_, with the electron density in the wake of the laser pulse still remaining well below *ρ*_*c*_. In this regime, the refractive index and the absorption coefficient of a solid, as well as the plasma skin layer and related parameters, can no longer be defined in terms of the slowly varying, field-phase-insensitive electron density. This difficulty can be addressed, as the analysis presented above show, by the electrodynamic response model expressed by Eqs (8, 20) above. When used jointly with the field-phase-resolving model of photoionization [Eqs (1, 5)], this model provides a framework for the description of the optical breakdown of solids by few-cycle laser pulses, which can be extended to describe the laser ablation of solids by few- and single-cycle optical field waveforms. The main physical limitations of this approach include the assumption that the dynamics of laser-induced ionization can be described with an assumption of a two-band dielectric and that the electron decoherence effects^30^ are negligible on the time scale of a few-cycle laser pulse. Unlike a standard two-band treatment, however, the dispersion profiles of the electron and conduction bands are not necessarily parabolic in our model, as Eq. (1) includes higher order Fourier harmonics of this dispersion profile, which can play a significant role in the nonlinear-optical response of a semiconductor^48–50^.

## Conclusion

To summarize, we have shown that a broadly accepted criterion of laser-induced breakdown in solids, defining the laser-breakdown threshold in terms of the laser fluence or laser intensity needed to generate a certain fraction of the critical electron density *ρ*_*c*_ within the laser pulse, fails in the case of high-intensity few-cycle laser pulses. Such laser pulses can give rise to subcycle oscillations of electron density *ρ* with peak *ρ* values well above *ρ*_*c*_ even when the total energy of the laser pulse is too low to induce a laser damage of material. The central idea of our approach is that, instead of the *ρ*/*ρ*_*c*_ ratio, the laser-breakdown threshold connects to the total laser energy coupled to the electron subsystem and subsequently transferred to the crystal lattice. With this approach, as the work presented here shows, predictions of the physical model start to converge to the available experimental data.
